# Fractional model of MHD blood flow in a cylindrical tube containing magnetic particles

**DOI:** 10.1038/s41598-021-04088-9

**Published:** 2022-01-10

**Authors:** Samina Majeed, Farhad Ali, Anees Imtiaz, Ilyas Khan, Mulugeta Andualem

**Affiliations:** 1 Department of Mathematics, City University of Science and Information Technology, Peshawar, 25000 Pakistan; 2 Department of Mathematics, College of Science Al-Zulfi, Majmaah University, Al-Majmaah, 11952 Saudi Arabia; 3 Department of Mathematics, Bonga University, Bonga, Ethiopia

**Keywords:** Chemical engineering, Mechanical engineering

## Abstract

In recent years, the use of magnetic particles for biomedicine and clinical therapies has gained considerable attention. Unique features of magnetic particles have made it possible to apply them in medical techniques. These techniques not only provide minimal invasive diagnostic tools but also transport medicine within the cell. In recent years, MRI, drug supply to infected tissue, Hyperthermia are more enhanced by the use of magnetic particles. The present study aims to observe heat and mass transport through blood flow containing magnetic particles in a cylindrical tube. Furthermore, the magnetic field is applied vertically to blood flow direction. The Caputo time fractional derivative is used to model the problem. The obtained partial fractional derivatives are solved using Laplace transform and finite Hankel transform. Furthermore, the effect of various physical parameters of our interest has also been observed through various graphs. It has been noticed that the motion of blood and magnetic particles is decelerated when the particle mass parameter and the magnetic parameter are increased. These findings are important for medicine delivery and blood pressure regulation.

## Introduction

The fluids like blood and polymer solutions are complex fluid and cannot be described by conventional Navier–Stokes’ equations. Such fluids are classified as non-Newtonian fluids^1^. Brinkman-type fluid is one of the types of non-Newtonian fluids. Brinkman type fluid is a kind of fluid that passes through a high permeable area^2^. Brinkman developed a model for fluid flow in the porous medium. It has enormous applications in science and engineering e.g. geohydrology, petroleum engineering, scientific study of soil, and manufacturing the products involving chemical processes^3^. In addition, Brinkman type fluid has massive applications in the medical field, e.g. Oxygen exchange in the blood through millions of alveoli in the lungs in capillaries, the procedure of blood dialysis in the artificial kidney, flow in blood oxygenation^4^. Ali et al*.*^5^ for the first time used Laplace transform technique to get the exact solution for the Brinkman type fluid model. The influence of radiative heat flux on Brinkman type fluid with the applied magnetic field is investigated Zakaria^6^. Blood flow in a cylindrical tube was examined by Saqib et al.^7^. In this study, blood is used as a Brinkman type fluid in this investigation. Magnetic particles are also injected into the bloodstream to investigate the effects of a perpendicularly applied magnetic field on blood and particle velocity. They found that raising values of the Brinkman parameter reduced the blood velocity. Ali et al*.*^8^examined the influence of thermal radiation on the natural convection flow of Brinkman type fluid by the use of an applied magnetic field.

Magnetic particles are metallic particles that are influenced by the magnetic field^8^. Magnetic particles has also the property of escalation of the thermal conductivity of working fluids when exposed to external magnetic field^9^. This property of magnetic particles makes it a good choice for researchers to use in the biomedical field^10^. Almost five decades ago magnetic particles were first time used for cancer treatment^11^. One of the characteristics of magnetic particles is their attraction to high magnetic flux density, which helps in drug targeting and biosepration^12^. Magnetic particles have the property of Hysteresis loss to the alternative magnetic field. This property is helpful in Hyperthermia^13^. Since the magnetic field is generated by magnetic particles that affects the surrounding local region. This property is used in magnetic resonance imaging (MRI)^14^. Other common uses of magnetic particles are gene transfer^15^, immunoassays^16^.

Keeping in mind the applications of magnetic particles in the biomedical field, various researchers are interested to discuss blood mixed with magnetic particles in different geometries under the application of magnetic field. Ali et al.^17^ briefly examined the role of magnetic particles for therapeutic purposes. Their model is based on blood flow with suspended magnetic particles and a magnetic field applied perpendicularly. Moreover, It is noticed that the motion of particles and blood can be regulated by using adequate use of magnetic field intensity. Furlani et al.^18^ used magnetic particles in the blood and formulated a mathematical model to introduce noninvasive magnetic targeting therapy. The results showed that this model is effective when the tumor is within few centimeters of the surface of the body. Grief et al.^19^ examined the effects of a perpendicularly applied magnetic field on blood flow with suspended magnetic particles. They noticed that by using magnetic particles, tumor treatment can be made more effective. In order to find an effective way to deliver localized genes effectively, Kilgus^20^ developed a model. It was noticed that the use of magnetic particles makes this process more effective. The model developed by Shit and Roy^21^ using magnetic particles in blood flow is helpful for the therapy of atherosclerosis and hypertension. Their investigation shows that the use of an external magnetic field is helpful to control blood flow. Mirza et al*.*^22^ explored the role of magnetic field for treatment of stenosed artery. They observed magnetized blood flow with suspended magnetic particles. During their investigation, strong variation in blood near the stenosed artery was observed.

Magnetic particles work as a heat source, scientists used this property to cure cancerous cells^23^. Choi and Eastman^24^ in their research work proved that adding a certain amount of metallic particles in fluid enhances the rate of heat transfer. This property of magnetic particles has attracted many researchers to conduct further studies. The effects of heat transfer on blood with suspended magnetic particles in a small capillary were observed by Ali et al.^25^. Khalid^26^ examined the influence of natural convection on flowing blood with suspended nanotubes and noticed that transfer of heat enhanced by the increase of carbon nanotube's volume fraction. Shah et al.^27^ analytically observed free convective blood flow. The effect of heat transmission on free convective fluid was investigated by Alsabery et al*.*^28^ in horizontal concentric annuls. Blood also plays a significant role in mass transfer to surrounding tissues. Researchers have also noticed experimentally that the existence of magnetic particles in blood improves mass transfer^29^. Tripathi et al.^30^ examined the behavior of two phase blood flow in a stenosed artery with combined effects of heat and mass transfer with an applied magnetic field.

Fractional calculus is almost three centuries old and is one of the exciting researches of applied analysis of sciences for modeling biological problems^31^. Fractional calculus shows hereditary and memory effects which are not possible by ordinary calculus^32^. To model any physical/biological problem, fractional calculus is more realistic than ordinary calculus. In recent years, many researchers have used fractional order calculus to model many biological problems. In order to observe the dynamic behavior of TB infection Rahman et al*.*^33^ developed a time fractional model. Their examination pointed how to diminish the contamination in human benig. They this investigation probed that fractional order derivative significantly analysis the model rather than classical derivative. Bansi et al.^34^ investigated blood flow in an artery using a fractional model. It was observed that fractional parameter is helpful to control temperature and motion of blood flow. Tabi et al.^35^ used time fractional model to describe the variation in motion of blood with embedded particles in a stenosed artery with an applied magnetic field. Their investigation showed that fractional parameter is more realistic to show the behavior of blood and particles. To investigate the frequency dependence of brain tissue, Kohandel et al.^36^ employed fractional calculus. By using fractional calculus, Ahmed et al.^37^ proposed a cancer model. This model showed that fractional order calculus is more effective to describe tumor immune system. To study the dynamics of tumor cells, Arfan et al.^38^ presented a time fractional model. The findings can be used to look into the dynamics of tumor cells, immune cells, and therapeutic reactions.

Keeping in mind all the above mentioned literature, a time fractional model has been established. The goal is to explore the impact of heat and mass transfer on blood with uniformly distributed magnetic particles flowing through a cylindrical tube. Moreover, blood flow is exposed to magnetic field. The Hankel and Laplace transformation are utilized to get the exact solution. The impacts of the various parameters are briefly described in several graphs.

## Problem formulation

**Figure 1:**
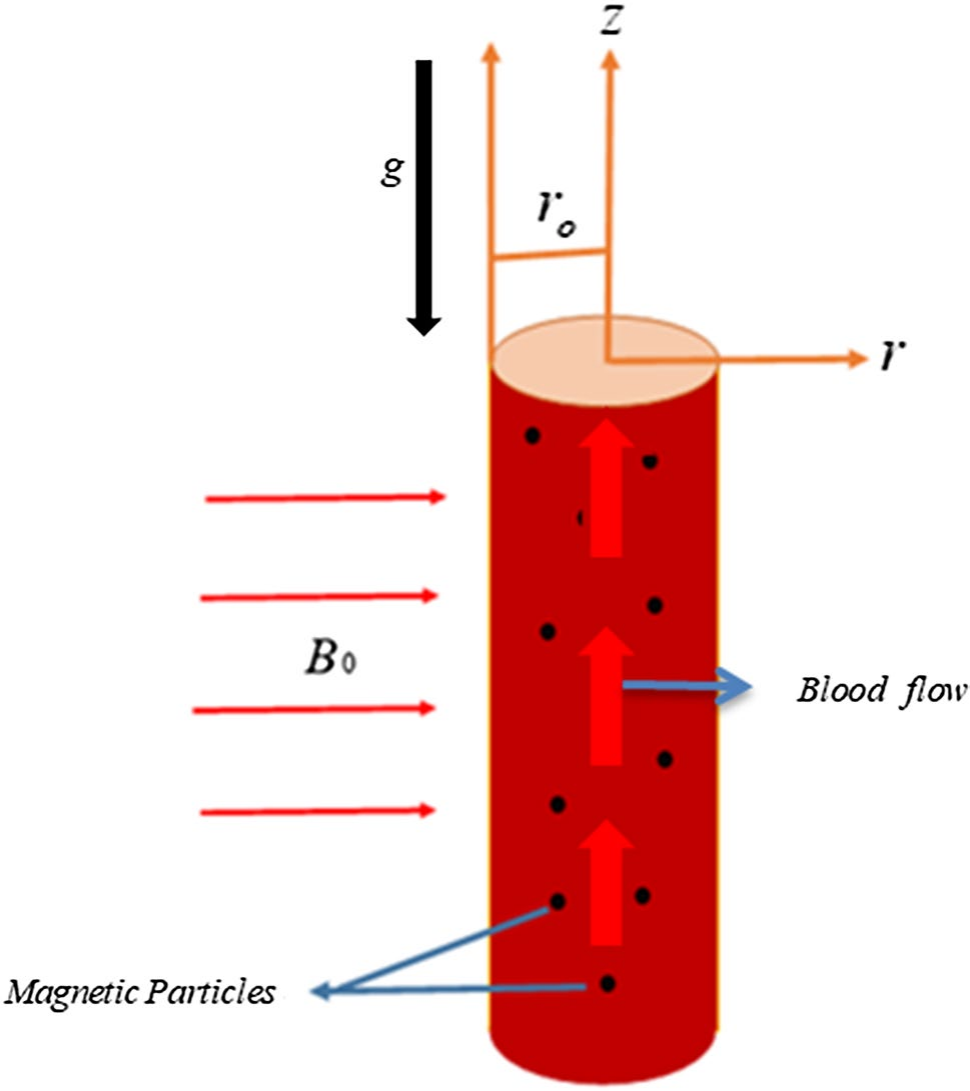
The geometry of the problem.

Consider unsteady blood flow in the axisymmetric circular cylinder of the radius *r*_0_ with suspended magnetic particles. In addition, Blood is considered to be Brinkman type fluid^10^. Blood involving magnetic particles is moving in the *z* direction, where the magnetic field is applied vertically to the direction of fluid flow as shown in Fig. 1. The intensity of the applied magnetic field is assumed to be strong as a result induced magnetic field is weak^39^. At *t* = 0, blood and magnetic particles are at rest. At *t* = 0^+^ fluid with suspended particles starts motion. Convective heat transfer and oscillating pressure gradient are responsible for fluid flow.

Governing equation for fluid motion is described by fluid Navier–Stoke’s equation, while particles motion is represented by Newton’s second law, and Maxwell's equation for electromagnetic field is defined as^40^

1 $$\left. \begin{gathered} \vec{\nabla } \times \vec{E} = - \frac{{\partial \vec{B}_{1} }}{\partial t}, \hfill \\ div\;\vec{B}_{1} = 0, \hfill \\ \vec{\nabla } \times \vec{B}_{1} = \mu_{0} \vec{J}, \hfill \\ \end{gathered} \right\},$$

According to Ohm’s law^41^

2 $$\vec{J} = \sigma 0\left( {\vec{E} + \mathop{V}\limits^{\rightharpoonup} \times \vec{B}} \right),$$

The electromagnetic force is defined as^42^

3 $$\mathop{F}\limits^{\rightharpoonup} em = \vec{J} \times \vec{B} = \sigma 0\left( {\vec{E} + \mathop{V}\limits^{\rightharpoonup} \times \vec{B}} \right) \times \vec{B} = - \sigma B0^{2} u\left( {r,t} \right)\mathop{k}\limits^{\rightharpoonup} .$$

where $\mathop{k}\limits^{\rightharpoonup}$ is unit vector in *z* direction.

### Momentum equation for blood

The velocity field for unsteady incompressible fluid flow mixed with magnetic in presence of pressure gradient and perpendicularly applied magnetic field in a cylindrical coordinate system ($r,\theta ,z$) is defined as^9,43^:

4 $$\vec{V} = u_{z} \left( {r,t} \right)\vec{k}.$$

Replacing $u_{z}$ by *u*, The fluid motion equations for incompressible Brinkman type fluid flow are:

5 $$\nabla \cdot \vec{V} = 0.$$

6 $$\rho \frac{{\partial \vec{V}}}{\partial t} + \rho \left( {\vec{V}.\nabla } \right)\vec{V} = div\;{\mathbf{T}} + (J \times B - I_{0} ).$$

Brinkman type fluid is modeled by the constitutive relation given as:

7 $${\mathbf{T}} = - {\text{P}}{\mathbf{I}} + \mu {\mathbf{A}}_{{1}}$$

where, $I$ is called identity tensor, and:

8 $${\mathbf{A}}_{{1}} = {\mathbf{L}} + {\mathbf{L}}^{{\mathbf{t}}} ,\;{\text{and}}\;{\mathbf{L}} = {\text{grad}}(\vec{V}).$$

We derive the following governing equations in components form as a result of our simplification:

9 $$\left. \begin{gathered} \frac{\partial u(r,t)}{{\partial t}} + \beta u(r,t) = - \frac{1}{\rho }\frac{\partial p}{{\partial z}} + \nu \left( {\frac{{\partial^{2} u(r,t)}}{{\partial r^{2} }} + \frac{1}{r}\frac{\partial u(r,t)}{{\partial r}}} \right) \hfill \\ \quad + \frac{KN}{\rho }\left( {u_{p} (r,t) - u(r,t)} \right) + g\beta_{T} (T - T\infty ) + g\beta_{C} (C - C\infty ) - \frac{1}{\rho }\sigma B0^{2} u(r,t) \hfill \\ \end{gathered} \right\},$$

the pressure gradient is taken in the following oscillating form^44^:

10 $$- \frac{\partial p}{{\partial z}} = \lambda 0 + \lambda 1\cos (\omega t)$$

Incorporating Eq. (10) in Eq. (9), we get

11 $$\left. \begin{gathered} \frac{\partial u(r,t)}{{\partial t}} + \beta u(r,t) = \frac{1}{\rho }\left( {\lambda 0 + \lambda 1\cos (\omega t)} \right) + \nu \left( {\frac{{\partial^{2} u(r,t)}}{{\partial r^{2} }} + \frac{1}{r}\frac{\partial u(r,t)}{{\partial r}}} \right) \hfill \\ \quad + \frac{KN}{\rho }\left( {u_{p} (r,t) - u(r,t)} \right) + g\beta_{T} (T - T\infty ) + g\beta_{C} (C - C\infty ) - \frac{1}{\rho }\sigma B_{0}^{2} u(r,t) \hfill \\ \end{gathered} \right\},$$

$\frac{KN}{\rho }\left( {u_{p} (r,t) - u(r,t)} \right)$ indicates the force due to the relative motion of fluid and magnetic particles.

For magnetic particles, the momentum equation is^45^:

12 $$m\frac{\partial up(r,t)}{{\partial t}} = K\left( {u(r,t) - up(r,t)} \right),$$

*m* is the mass of magnetic particles.

The equation for energy and mass concentration is as follows^46^:

13 $$\rho c_{p} \frac{\partial T}{{\partial t}} = k\left( {\frac{{\partial^{2} T}}{{\partial r^{2} }} + \frac{1}{r}\frac{\partial T}{{\partial r}}} \right),\,\,\,\,\,\,\,\,t > 0,\,\,r \in (0,r_{0} )$$

14 $$\frac{\partial C}{{\partial t}} = D\left( {\frac{{\partial^{{^{2} }} C}}{{\partial r^{2} }} + \frac{1}{r}\frac{\partial C}{{\partial r}}} \right),\,\,\,\,\,\,\,\,\,\,\,\,\,\,t > 0,\,\,r \in (0,r_{0} )$$

Subjected to the following initial and boundary conditions

15 $$\left. \begin{gathered} u(r,0) = 0,\quad \quad u_{p} (r,0) = 0,\quad \quad T(r,0) = T_{\infty } ,\quad \quad C(r,0) = C_{\infty } \hfill \\ u(r_{0} ,t) = H(t)u_{0} ,\quad \quad \quad \quad up(r_{0} ,t) = H(t)u_{0} \left[ {1 - e^{{ - \frac{K}{m}t}} } \right], \hfill \\ \,\,T(r_{0} ,t) = T_{w} ,\quad \quad C(r_{0} ,t) = Cw,\quad \quad \left. {\frac{\partial u}{{\partial r}}} \right|_{r = 0} = 0 \hfill \\ \end{gathered} \right\},$$

Introducing the dimensionless variables16

The given dimensionless equations obtained17

18 $$\frac{\partial g(\xi ,\tau )}{{\partial \tau }} = Pm\left( {f(\xi ,\tau ) - g(\xi ,\tau )} \right),$$

19 $$\frac{\partial \Theta (\xi ,\tau )}{{\partial \tau }} = \frac{1}{\Pr }\left( {\frac{{\partial^{2} \Theta (r1,\tau )}}{{\partial \xi^{2} }} + \frac{1}{\xi }\frac{\partial \Theta (\xi ,\tau )}{{\partial \xi }}} \right),$$

20 $$\frac{\partial \Phi (\xi ,\tau )}{{\partial \tau }} = \frac{1}{Sc}\left( {\frac{{\partial^{2} \Phi (r1,\tau )}}{{\partial \Phi^{2} }} + \frac{1}{\xi }\frac{\partial \phi (\xi ,\tau )}{{\partial \xi }}} \right),$$

where, $$\Pr = \frac{{\mu \,c_{p} }}{k},\,\,\,\,\,\,Sc = \frac{\nu }{D},$$

Subjected to the following IC and BCs:

21 $$\left. \begin{gathered} f(\xi ,0) = 0,\quad g(\xi ,0) = 0,\quad \Theta (\xi ,0) = 0,\quad \Phi (\xi ,0) = 0, \hfill \\ f(1,\tau ) = 1,\quad g(1,\tau ) = \left[ {1 - e^{ - bt} } \right],\quad \Theta (1,t) = 1,\quad \Phi (1,t) = 1,\quad \hfill \\ \left. {\frac{\partial f(\xi ,\tau )}{{\partial \xi }}} \right|_{\xi = 0} = 0, \hfill \\ \end{gathered} \right\},$$

where

22 $$\left. \begin{gathered} Pc = \frac{{KNr_{0}^{2} }}{\mu },\quad M0 = M - \beta_{1} ,\quad Pm = \frac{{Kr_{0}^{2} }}{\nu },\quad \beta 1 = \frac{{\beta r_{0}^{2} }}{\nu }, \hfill \\ \,Gr = \frac{{gr_{0}^{2} \beta_{T} \left( {Tw - T\infty } \right)}}{{u_{0} \mu }},\quad b = \frac{{Kr_{0}^{2} }}{m\nu }, \hfill \\ Gm = \frac{{gr_{0}^{2} \beta_{C} \left( {Cw - C\infty } \right)}}{{u_{0} \mu }},\quad M = \frac{{\sigma B_{0} r_{0}^{2} }}{\mu }, \hfill \\ \end{gathered} \right\},$$

Taking the Caputo time fractional derivative of Eqs. (17) to (20) we obtain:23

24 $$D_{t}^{\alpha } g(\xi ,\tau ) = Pm\left( {f(\xi ,\tau ) - g(\xi ,\tau )} \right),$$

25 $$D_{t}^{\alpha } \Theta (\xi ,\tau ) = \frac{1}{\Pr }\left( {\frac{{\partial^{2} \Theta (\xi ,\tau )}}{\partial \xi } + \frac{1}{\xi }\frac{\partial \Theta (\xi ,\tau )}{{\partial \xi }}} \right),$$

26 $$D_{t}^{\alpha } \Phi (\xi ,\tau ) = \frac{1}{Sc}\left( {\frac{{\partial^{2} \Phi (\xi ,\tau )}}{\partial \xi } + \frac{1}{\xi }\frac{\partial \Phi (\xi ,\tau )}{{\partial \xi }}} \right),$$

where, Definition of Caputo time fractional derivative is as follows^47^:

27 $$\begin{gathered} D_{t}^{\alpha } f\left( {r,t} \right) = \left\{ \begin{gathered} \frac{1}{\Gamma (1 - \alpha )}\int\limits_{0}^{t} {\frac{{f^{/} (\tau )}}{{(t - \tau )^{\alpha } }}} d(\tau );\,\,\,\,\,\,\,\,\,\,\,0 < \alpha < 1, \hfill \\ \,\frac{\partial f(t)}{{\partial t}}\,\,\,\,\,\,\,\,\,\,\,\,\,\,\,\,\,\,\,\,\,\,\,\,\,\,\,\,\,\,\,\,\,\,\,\,\,\,\,\,\,\,\,\,\,\,\,\,\,\,\,\,\,\alpha = 1.\,\,\,\,\,\,\,\,\,\,\,\, \hfill \\ \end{gathered} \right. \hfill \\ \, \hfill \\ \end{gathered}$$

## The solution of the problem

In order to get the solutions for velocity field, Temperature field, and concentration profile, FHT and LT are utilized defined as under^48,49^:

28 $$\left. \begin{gathered} L\{ f(r,t)\} (\ell ) = \overline{f}(\ell ) = \int\limits_{0}^{\infty } {f(r,t)e^{ - \ell t} dt,} \hfill \\ H\{ \overline{f}(r,\ell )\} (\varepsilon n) = \overline{f}H(\varepsilon n,\ell ) = \int\limits_{0}^{1} {r\overline{f}(r,\ell )J0(r\varepsilon n)dr.} \hfill \\ \end{gathered} \right\}$$

where $J_{0} \left( {\varepsilon_{n} } \right)$ is Bessel’s function of the first kind of order zero. $\varepsilon_{n}$ are positive roots of equation $J_{0} \left( x \right) = 0$.

### Temperature field calculation

Equations derived by applying LT to Eqs. (25) and (21) are:

29 $$\ell^{\alpha } \overline{\Theta }(\xi ,\ell ) = \frac{1}{\Pr }\left( {\frac{{d^{2} \overline{\Theta }(\xi ,\ell )}}{{d\xi^{2} }} + \frac{1}{\xi }\frac{{d\overline{\Theta }(\xi ,\ell )}}{d\xi }} \right),$$

30 $$\overline{\Theta }(1,\ell ) = \frac{1}{\ell },$$

$\overline{\Theta }(\xi ,\ell )$ of is Laplace transform of $\Theta (\xi ,t)$, and $\ell$ is called the transformation variable.

Equations derived by applying FHT to Eq. (29) and substituting values from Eq. (30), are as follows:

31 $$\overline{\Theta }_{H} (\varepsilon_{1n} ,\ell ) = \frac{{\varepsilon_{1n} \,J(\varepsilon_{1n} )}}{\Pr }\frac{1}{{\ell \left( {\ell^{\alpha } + a_{1} } \right)}},$$

where $$\frac{{\varepsilon_{1n}^{2} }}{\Pr } = a_{1}$$.

$\overline{\Theta }_{H} \left( {\varepsilon_{1n} ,\,\ell } \right)$ is the HT of $\overline{\Theta }\left( {r,\,\ell } \right)$ simplified form of Eq. (31) is:

32 $$\overline{\Theta }_{H} (\varepsilon_{1n} ,\ell ) = \frac{{J_{1} (\varepsilon_{1n} )}}{{\varepsilon_{1n} }}\frac{1}{\ell } - \frac{{J_{1} (\varepsilon_{1n} )}}{{\varepsilon_{1n} }}\frac{{\ell^{ - (1 - \alpha )} }}{{\ell^{\alpha } + a_{1} }},$$

Applying inverse Laplace transform by using Lorenzo and Hartley’s $R_{\chi ,\upsilon } \left( { - \gamma^{*} ,\Im } \right)$ functions^50^ to Eq. (32)

33 $$\Theta_{H} (\varepsilon_{1n} ,\tau ) = \frac{{J_{1} (\varepsilon_{1n} )}}{{\varepsilon_{1n} }} - \frac{{J_{1} (\varepsilon_{1n} )}}{{\varepsilon_{1n} }}R_{(\alpha ,1 - \alpha )} (\tau , - a_{1} ),$$

where

34 $$R_{\chi ,\upsilon } \left( { - \gamma^{*} ,\Im } \right) = L^{ - 1} \left( {\frac{{\ell^{ - \upsilon } }}{{\ell^{\chi } + \gamma^{*} }}} \right) = \sum\limits_{n = 0}^{\infty } {\frac{{\left( { - \gamma^{*} } \right)^{n} \Im^{{\left( {n + 1} \right)\gamma^{*} - 1 - \upsilon }} }}{{\Gamma \{ \left( {n + 1} \right)\gamma^{*} - \upsilon \} }}} ,$$

Taking the inverse HT of Eq. (26), the obtained equation is:

35 $$\Theta (\xi ,\tau ) = 1 - 2\sum\limits_{n = 1}^{\infty } {\frac{{J_{0} (\xi \varepsilon_{1n} )}}{{\varepsilon_{1n} \,\,J_{1} (\varepsilon_{1n} )}}R_{(\alpha ,1 - \alpha )} } ( - a_{1} ,\tau ),$$

### Calculation fluid concentration

Taking Laplace transform to Eqs. (27) and (21) obtained equations are:

36 $$\ell^{\alpha } \overline{\Phi } (\xi ,\ell ) = \frac{1}{Sc}\left( {\frac{{d^{2} \overline{\Phi } (\xi ,\ell )}}{{d\xi^{2} }} + \frac{1}{\xi }\frac{{d\overline{\Phi } (\xi ,\ell )}}{d\xi }} \right),$$

37 $$\overline{\Phi } (1,\ell ) = \frac{1}{\ell },$$

$\overline{\Phi } (\xi ,\ell )$ is Laplace transform of $\overline{\Phi } (\xi ,t),$ where $\ell$ denotes transformation variable.

The equations derived by applying FHT to Eq. (28) and substituting results from Eq. (37) are::

38 $$\overline{\Phi }_{H} (\varepsilon_{1n} ,\ell ) = \frac{{\varepsilon_{1n} J(\varepsilon_{1n} )}}{Sc}\frac{1}{{\ell \left( {\ell^{\alpha } + a_{2} } \right)}},$$

where $$\frac{{\varepsilon_{1n}^{2} }}{Sc} = a_{2} ,$$

$\overline{\Phi } \left( {\varepsilon_{1n} ,\,\ell } \right)$ shows Hankel transform of $\overline{\Phi } \left( {r,\,\ell } \right)$. Equation (38) reduces to:

39 $$\overline{\Phi }_{H} (\varepsilon_{1n} ,\ell ) = \frac{{J_{1} (\varepsilon_{1n} )}}{{\varepsilon_{1n} }}\frac{1}{\ell } - \frac{{J_{1} (\varepsilon_{1n} )}}{{\varepsilon_{1n} }}\frac{{\ell^{ - (1 - \alpha )} }}{{\ell^{\alpha } + a_{2} }},$$

Lorenzo and Hartley’s $R_{\chi ,\upsilon } \left( { - \lambda^{*} ,\Im } \right)$ functions^50^ to Eq. (39) to get inverse LT:

40 $$\Phi_{H} (\varepsilon_{1n} ,\tau ) = \frac{{J_{1} (\varepsilon_{1n} )}}{{\varepsilon_{1n} }} - \frac{{J_{1} (\varepsilon_{1n} )}}{{\varepsilon_{1n} }}R_{(\alpha ,1 - \alpha )} (\tau , - a_{2} ),$$

Taking the inverse Henkel transform of Eq. (40), we obtain:

41 $$\Phi (\xi ,\tau ) = 1 - 2\sum\limits_{n = 1}^{\infty } {\frac{{J_{0} (\xi \varepsilon_{1n} )}}{{r_{1n} \,J_{1} (\varepsilon_{1n} )}}R_{(\alpha ,1 - \alpha )} } ( - a_{2} ,\tau ),$$

### Calculation for blood flow

Taking LT of Eqs. (23) and (24) we obtain:42

43 $$\overline{g}(\xi ,\ell ) = \frac{{\overline{f}(\xi ,\ell )}}{{Pm\,\ell^{\alpha } + 1}},$$

Applying the HT to Eqs. (42) and (43) we get:44

45 $$\overline{g}_{H} \left( {\varepsilon_{1n} ,q} \right) = \frac{1}{Pm}\left( {\frac{1}{{\ell^{\alpha } + \frac{1}{Pm}}}} \right)\overline{f}_{H} \left( {\varepsilon_{1n} ,\ell } \right),$$

where,

46 $$\left. \begin{gathered} \int\limits_{0}^{1} {\left( {\frac{{\partial^{2} \overline{f}(\xi ,\ell )}}{\partial \xi } + \frac{1}{\xi }\frac{{\partial \overline{f}(\xi ,\ell )}}{\partial \xi }} \right)dr_{1} = } - \varepsilon_{1n}^{2} .\overline{f}_{H} (r_{1n} ,\ell ) + \varepsilon_{1n} \,J_{1} (\varepsilon_{1n} )\overline{f}(1,\ell ), \hfill \\ \overline{f}(1,\ell ) = \frac{1}{\ell }, \hfill \\ \end{gathered} \right\},$$

Simplifying Eq. (44) leads to:

47 $$\left( {\frac{{\ell^{2\alpha } + \Upsilon_{0} \ell^{\alpha } + \Upsilon_{1} }}{{\ell^{\alpha } + \Upsilon_{2} }}} \right)\overline{f}_{H} (\varepsilon_{1n} ,\ell ) = \overline{F}_{0n} (\ell ) + \frac{{J_{1} (\varepsilon_{1n} )}}{\ell } + Gr\overline{\Theta }_{H} (\varepsilon_{1n} ,\ell ) + Gm\overline{{\Phi_{H} }} (\varepsilon_{1n} ,\ell ),$$

where,48

After simplification Eq. (38) reduces to:

49 $$\overline{f}_{H} (\varepsilon_{1n} ,\ell ) = \left\{ \begin{gathered} \frac{{\varepsilon_{1n} \,J_{1} (\varepsilon_{1n} )}}{\ell } + \overline{F}_{0n} (\ell )\frac{{J_{1} (\varepsilon_{1n} )}}{{\varepsilon_{1n} }} + \hfill \\ Gr\left( {\left( {\frac{1}{\ell } - \frac{{\ell^{\alpha - 1} }}{{\ell^{\alpha } + a_{1} }}} \right)\frac{{J_{1} (\varepsilon_{1n} )}}{{\varepsilon_{1n} }}} \right) + \hfill \\ Gm\left( {\left( {\frac{1}{\ell } - \frac{{\ell^{\alpha - 1} }}{{\ell^{\alpha } + a_{2} }}} \right)\frac{{J_{1} (\varepsilon_{1n} )}}{{\varepsilon_{1n} }}} \right) \hfill \\ \end{gathered} \right\}\left( {\frac{{\ell^{\alpha } + \Upsilon_{2} }}{{(\ell^{\alpha } + \Upsilon_{3} )(\ell^{\alpha } + \Upsilon_{4} )}}} \right),$$

Equation (39) can be written as:50where

51 $$\left. \begin{gathered} \Upsilon 3 = \frac{{\Upsilon 0 + \sqrt {\Upsilon 0 - 4\Upsilon 1} }}{2},\quad \quad \Upsilon 4 = \frac{{\Upsilon 0 - \sqrt {\Upsilon 0 - 4\Upsilon 1} }}{2},\quad \quad \Upsilon 5 = \Upsilon 3 + \Upsilon {4}, \hfill \\ \,\Upsilon 6 = \Upsilon 3\Upsilon {4},\quad \quad \Upsilon 7 = \varepsilon_{1n}^{2} - \Upsilon 5,\quad \quad \Upsilon 8 = \varepsilon_{1n}^{2} \Upsilon 2 - \Upsilon 6, \hfill \\ \wp 0 = \frac{{\Upsilon 8 - \Upsilon 7\Upsilon 3 - \Upsilon_{3}^{2} }}{{\Upsilon 3 - \Upsilon {4}}},\quad \quad \wp 1 = \frac{{ - \Upsilon 8 + \Upsilon 7\Upsilon {4 + }\Upsilon_{4}^{2} }}{{(\Upsilon 3 - \Upsilon {4)}}},\quad \quad \wp 2 = \frac{\Upsilon 1 - \Upsilon 2}{{\Upsilon 3 - \Upsilon {4}}}, \hfill \\ \wp 3 = \frac{\Upsilon 2 - \Upsilon 4}{{\Upsilon 3 - \Upsilon {4}}},\quad \quad \wp 4 = \frac{a1}{{a1 - \Upsilon 3}},\quad v\wp 5 = \frac{\Upsilon 3}{{a1 - \Upsilon 3}}, \hfill \\ \wp 6 = \frac{a1}{{a1 - \Upsilon {4}}},\quad \quad \wp 7 = \frac{{\Upsilon {4}}}{{a1 - \Upsilon {4}}},\wp 8 = \wp 4 + \wp 6, \hfill \\ \wp 9 = \wp 2 + \wp 5,\quad \quad \wp 10 = \wp 3 + \wp 7,\quad \quad \wp 11 = \frac{a2}{{a2 - k3}}, \hfill \\ \wp 12 = \frac{\Upsilon 3}{{a2 - \Upsilon 3}},\quad \quad \wp 13 = \frac{a2}{{a2 - \Upsilon {4}}},\quad \quad \wp 14 = \frac{{\Upsilon {4}}}{{a2 - \Upsilon {4}}}, \hfill \\ \wp 15 = \wp 11 + \wp 13,\quad \quad \wp 16 = \wp 2 + \wp 12,\quad \quad \wp 17 = \wp 3 + \wp 14, \hfill \\ \end{gathered} \right\},$$

Applying inverse LT to Eq. (51) we get:

52 $$f_{H} \left( {\varepsilon_{1n} ,\tau } \right) = \frac{{J_{1} (\varepsilon_{1n} )}}{{\varepsilon_{1n} }} - \frac{{J_{1} (\varepsilon_{1n} )}}{{\varepsilon_{1n} }}\left\{ \begin{gathered} 1 + N_{1} \,R\alpha , - 1( - \Upsilon 3,\tau ) + N_{2} \,R_{\alpha , - 1} ( - \Upsilon_{4} ,\tau ) \hfill \\ + N_{3} \,\cos (\omega \,t)\,*F_{\alpha } ( - \Upsilon_{3} ,\tau ) \hfill \\ + N_{4} \,\,\cos (\omega \,t)*F_{\alpha } ( - \Upsilon_{4} ,\tau ) \hfill \\ + N_{5} \,R_{\alpha , - 1} ( - a_{1} ,\tau ) + N_{6} R_{\alpha , - 1} ( - a_{2} ,\tau ) \hfill \\ \end{gathered} \right\},$$

53 $$g_{H} \left( {\varepsilon_{1n} ,\tau } \right) = \frac{1}{Pm}F_{\alpha } \left( { - \frac{1}{Pm},\tau } \right)*f_{H} \left( {\varepsilon_{1n} ,\tau } \right),$$

where

$$F_{\chi } \left( { - \lambda^{*} ,\Im } \right) = \sum\limits_{n = 0}^{\infty } {\frac{{\left( { - \gamma^{*} } \right)^{n} \Im^{{\left( {n + 1} \right)\gamma^{*} - 1}} }}{{\Gamma \{ \left( {n + 1} \right)\chi \} }}} = L^{ - 1} \left( {\frac{1}{{s^{\chi } + \gamma^{*} }}} \right),$$

is Robotnov and Hartley’s function^50^

Applying inverse FHT of Eq. (32) reduces to:

54 $$\,f\left( {\xi ,\tau } \right) = 1 - 2\sum\limits_{n = 1}^{\infty } {\frac{{J_{0} \left( {\xi \varepsilon_{1n} } \right)}}{{\varepsilon_{1n} \,J_{1} \left( {\varepsilon_{1n} } \right)}}\left\{ \begin{gathered} 1 + \overset{\lower0.5em\hbox{$\smash{\scriptscriptstyle\smile}$}}{N}_{1} \,R_{\alpha , - 1} ( - \Upsilon_{3} ,\tau ) + \overset{\lower0.5em\hbox{$\smash{\scriptscriptstyle\smile}$}}{N} 2\,\,R_{\alpha , - 1} ( - \Upsilon_{4} ,\tau ) \hfill \\ + \overset{\lower0.5em\hbox{$\smash{\scriptscriptstyle\smile}$}}{N}_{3} \,\,\cos (\omega \,t)\,*F_{\alpha } ( - \Upsilon_{3} ,\tau ) \hfill \\ + \overset{\lower0.5em\hbox{$\smash{\scriptscriptstyle\smile}$}}{N}_{4} \,\,\cos (\omega \,t)*F_{\alpha } ( - \Upsilon_{4} ,\tau ) \hfill \\ + \overset{\lower0.5em\hbox{$\smash{\scriptscriptstyle\smile}$}}{N}_{5} \,R_{\alpha , - 1} ( - a_{1} ,\tau ) + \overset{\lower0.5em\hbox{$\smash{\scriptscriptstyle\smile}$}}{N} 6\,R_{\alpha , - 1} ( - a_{2} ,\tau ) \hfill \\ \end{gathered} \right\}} ,$$

55 $$g\left( {\xi ,\tau } \right) = \frac{1}{Pm}F_{\alpha } \left( { - \frac{1}{Pm},\tau } \right)*f\left( {\xi ,\tau } \right),$$

where56

### Limiting cases

**Case-I**: taking $$\alpha = 1$$.

When $\alpha \to 1$, the Robotnov and Hartley’s Lorenzo and Hartley’s and function become^51^

$$R_{1, - 1} \left( { - \gamma^{*} ,\Im } \right) = L^{ - 1} \left( {\frac{{\ell^{ - 1} }}{{\ell + \gamma^{*} }}} \right) = \frac{{1 - e^{{\gamma^{*} \Im }} }}{{\gamma^{*} }} = \sum\limits_{n = 0}^{\infty } {\frac{{\left( { - \gamma^{*} } \right)^{n} \Im^{n + 1} }}{k!}} ,$$

$$R_{1,0} = e^{{ - }{\gamma^{*} \Im }}$$

$$F1\left( { - \gamma^{*} ,\Im } \right) = L^{ - 1} \left( {\frac{1}{{s + \gamma^{*} }}} \right) = \sum\limits_{n = 0}^{\infty } {\frac{{\left( { - \gamma^{*} } \right)^{n} \Im^{n} }}{k!}} = e^{{ - }{\gamma^{*} \Im }} ,$$

Equations (27), (33), (43) and (44) reduces to

57 $$\Theta (\xi ,\tau ) = 1 - 2\sum\limits_{n = 1}^{\infty } {\frac{{J_{0} (\xi \varepsilon_{1n} )}}{{\varepsilon_{1n} \,\,J_{1} (\varepsilon_{1n} )}}e^{{ - a_{1} \tau }} ,}$$

58 $$\Phi (\xi ,\tau ) = 1 - 2\sum\limits_{n = 1}^{\infty } {\frac{{J_{0} (\xi \varepsilon_{1n} )}}{{\varepsilon_{1n} \,\,J_{1} (\varepsilon_{1n} )}}e^{{ - \alpha_{2} \tau }} ,}$$

59 $$f\left( {\xi ,\tau } \right) = 1 - 2\sum\limits_{n = 1}^{\infty } {\frac{{J_{0} \left( {\xi \varepsilon_{1n} } \right)}}{{\varepsilon_{1n} \,J_{1} \left( {\varepsilon_{1n} } \right)}}\left\{ \begin{gathered} 1 + \overset{\lower0.5em\hbox{$\smash{\scriptscriptstyle\smile}$}}{N}_{01} \left( {1 - e^{{\Upsilon_{3} \tau }} } \right) + \overset{\lower0.5em\hbox{$\smash{\scriptscriptstyle\smile}$}}{N} 02\left( {1 - e^{{\Upsilon_{4} \tau }} } \right)) \hfill \\ + \overset{\lower0.5em\hbox{$\smash{\scriptscriptstyle\smile}$}}{N}_{03} \,\,\left( {e^{{ - \Upsilon_{3} \tau }} + \cos (\omega t) + w0\sin (\omega t)} \right) \hfill \\ + \overset{\lower0.5em\hbox{$\smash{\scriptscriptstyle\smile}$}}{N}_{04} \left( {e^{{ - \Upsilon_{4} \tau }} + \cos (\omega t) + w1\sin (\omega t)} \right) \hfill \\ + \overset{\lower0.5em\hbox{$\smash{\scriptscriptstyle\smile}$}}{N}_{05} \,\left( {1 - e^{{a_{1} \tau }} } \right) + \overset{\lower0.5em\hbox{$\smash{\scriptscriptstyle\smile}$}}{N} 06\,\left( {1 - e^{{\alpha_{2} \tau }} } \right). \hfill \\ \end{gathered} \right\}} ,$$

60 $$g\left( {r_{1} ,\tau } \right) = \frac{1}{Pm}e^{{ - \frac{1}{Pm}\tau }} *f\left( {r_{1} ,\tau } \right),$$

where,

61 $$\left. \begin{gathered} \overset{\lower0.5em\hbox{$\smash{\scriptscriptstyle\smile}$}}{N}_{01} = \frac{{\overset{\lower0.5em\hbox{$\smash{\scriptscriptstyle\smile}$}}{N}_{1} }}{{\Upsilon_{3} }},\quad \overset{\lower0.5em\hbox{$\smash{\scriptscriptstyle\smile}$}}{N} 02 = \frac{{\overset{\lower0.5em\hbox{$\smash{\scriptscriptstyle\smile}$}}{N}_{2} }}{{\Upsilon_{4} }},\quad N_{03} = \frac{{\overset{\lower0.5em\hbox{$\smash{\scriptscriptstyle\smile}$}}{N}_{3} }}{{\Upsilon_{3} }}\left( {\frac{{\Upsilon_{3}^{2} }}{{\Upsilon_{3}^{2} - w^{2} }}} \right),\quad \overset{\lower0.5em\hbox{$\smash{\scriptscriptstyle\smile}$}}{N}_{04} = \frac{{\overset{\lower0.5em\hbox{$\smash{\scriptscriptstyle\smile}$}}{N}_{4} }}{{\Upsilon_{4} }}\left( {\frac{{\Upsilon_{4}^{2} }}{{\Upsilon_{4}^{2} - w^{2} }}} \right), \hfill \\ w_{0} = \frac{w}{{\Upsilon_{3}^{2} }},\quad w1 = \frac{w}{{\Upsilon_{4}^{2} }},\quad \overset{\lower0.5em\hbox{$\smash{\scriptscriptstyle\smile}$}}{N}_{05} = \frac{{N_{5} }}{{a_{1} }},\quad \overset{\lower0.5em\hbox{$\smash{\scriptscriptstyle\smile}$}}{N}_{06} = \frac{{N_{6} }}{{a_{2} }} \hfill \\ \end{gathered} \right\}.$$

The limiting solution (57) and (58) is quite in agreement with the published work Shah et al.^27^.

**Case-II**: For $Gm = 0,$ the obtained general solution (54) reduces to

62 $$f\left( {\xi ,\tau } \right) = 1 - 2\sum\limits_{n = 1}^{\infty } {\frac{{J_{0} \left( {r_{1} \varepsilon_{1n} } \right)}}{{\varepsilon_{1n} J_{1} \left( {\varepsilon_{1n} } \right)}}\left\{ \begin{gathered} 1 + N_{07} \,R_{\alpha , - 1} ( - \Upsilon_{3} ,\tau ) + N_{08} \,\,R_{\alpha , - 1} ( - \Upsilon_{4} ,\tau ) \hfill \\ + N_{3} \,\,\cos (\omega t)*F_{\alpha } ( - \Upsilon_{3} ,\tau ) \hfill \\ + N_{4} \,\,\cos (\omega t)*F_{\alpha } ( - \Upsilon_{4} ,\tau ) \hfill \\ + N_{5} \,R_{\alpha , - 1} ( - a_{1} ,\tau ) \hfill \\ \end{gathered} \right\}} .$$

63

The limiting solution (62) is quite in agreement with the published work Ali et al. ^52^.

## Graphical results and discussion

**Figure 2:**
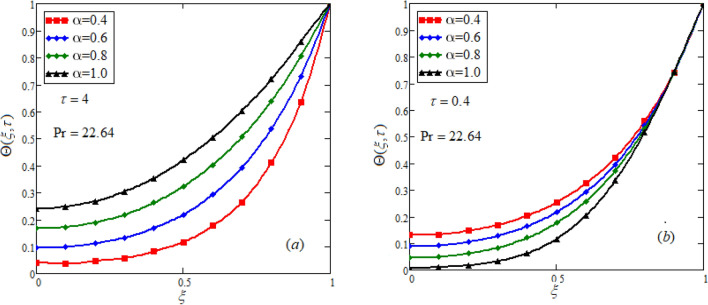
Impact of $\alpha$ on temperature field for long and short time.

**Figure 3:**
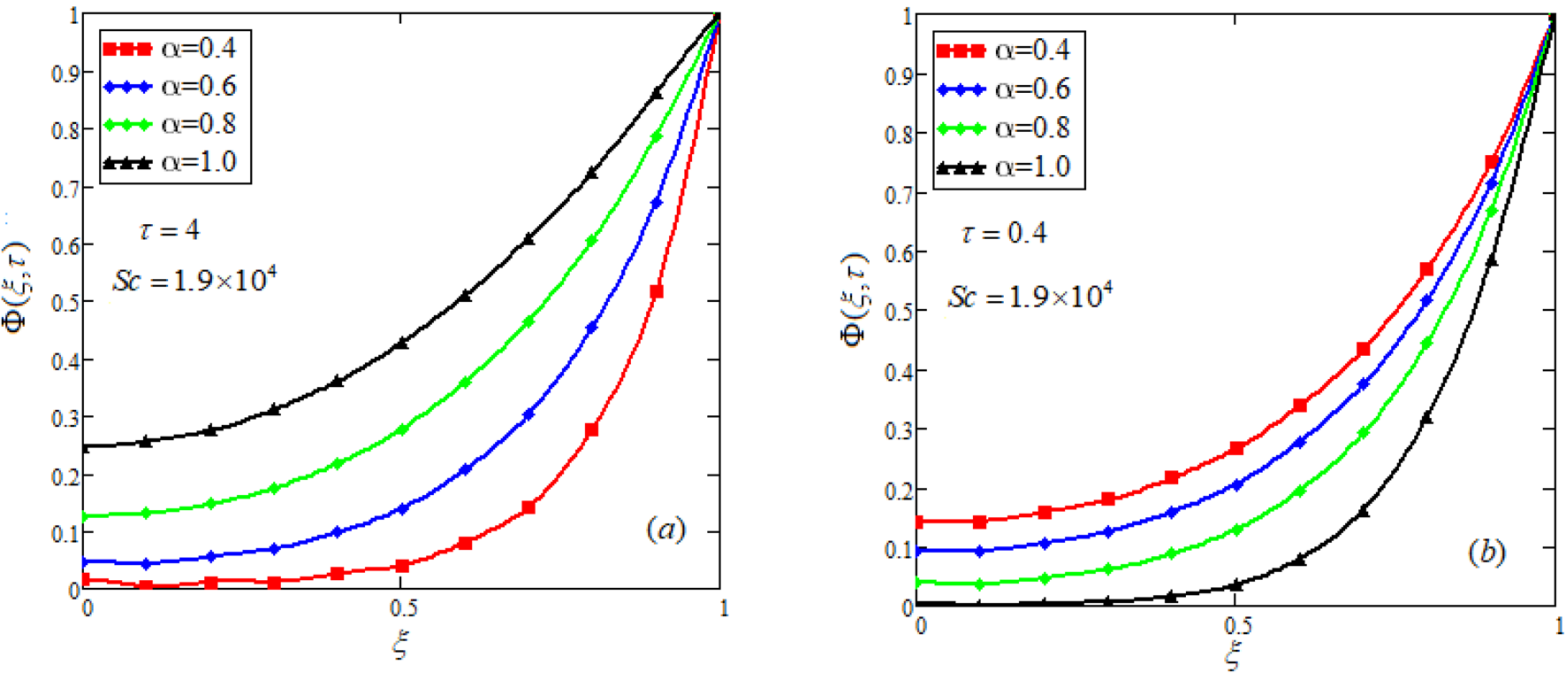
Impact of $\alpha$ on concentration profile for long and short time. $M = 0.5,Pm = 0.5,\alpha = 0.5,Gm = 0.5,M = 0.5,Pc = 0.5.$

**Figure 4:**
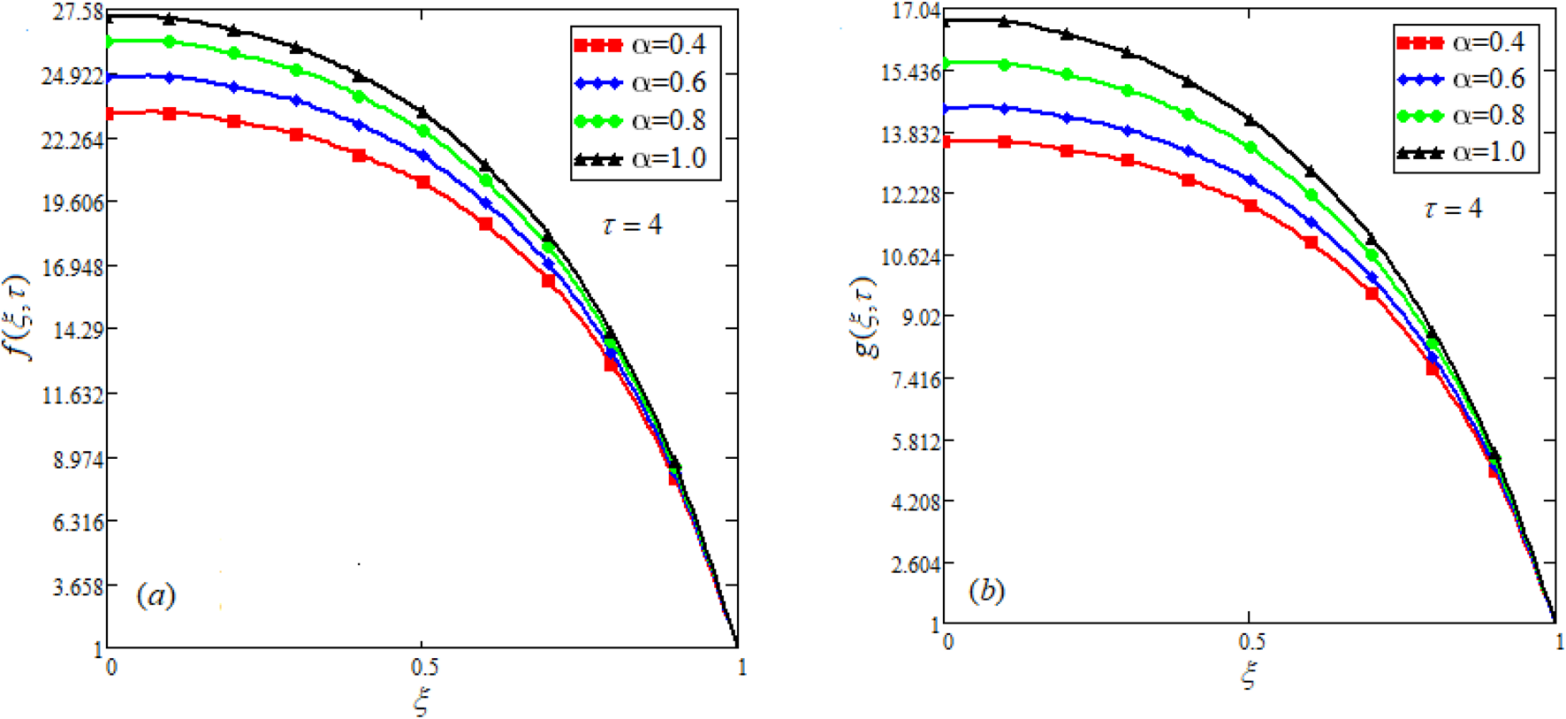
Effect of $\alpha$ on blood and particle velocity for $\tau > 1$. $Pm = 0.8,\beta = 0.5,M = 0.5,Pc = 0.5,Gm = 0.5.$

**Figure 5:**
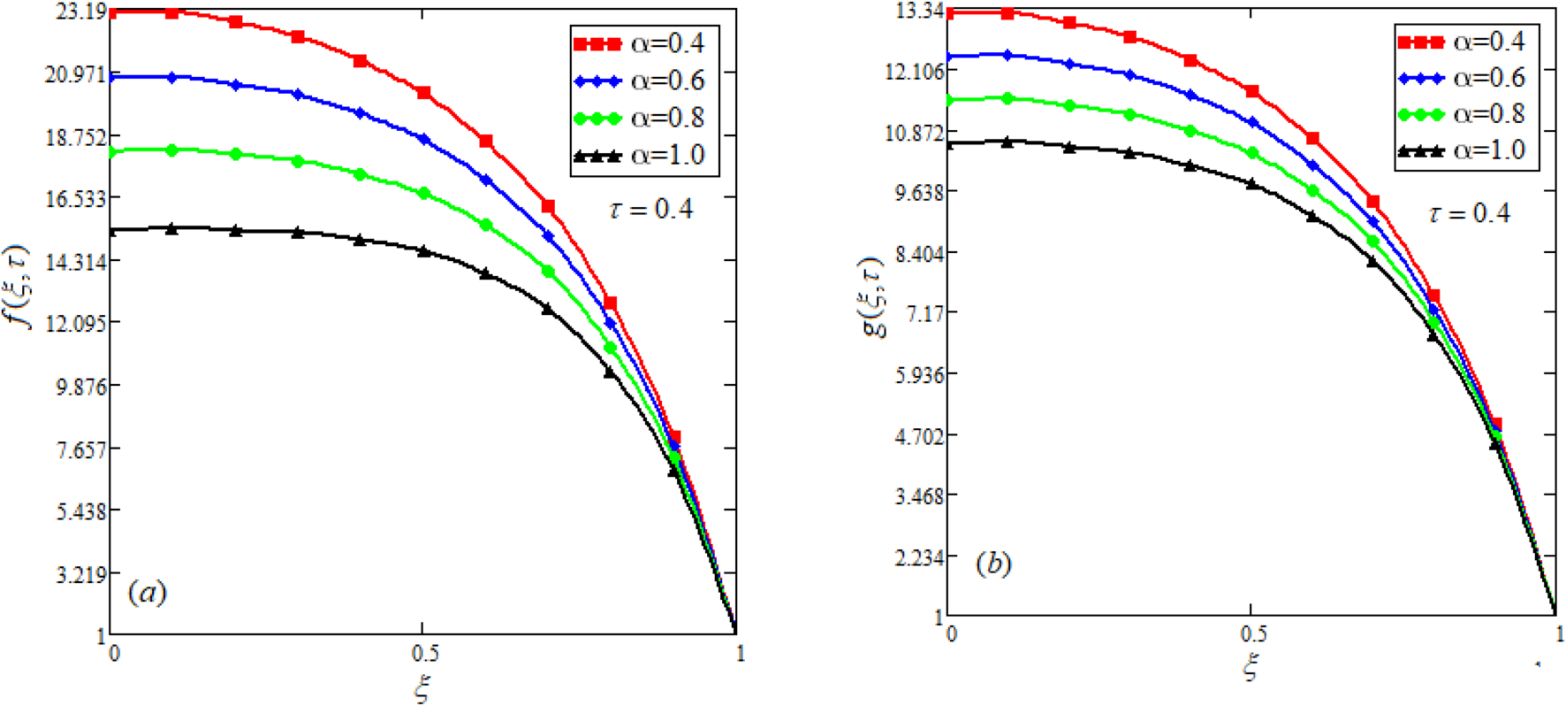
Effect of $\alpha$ on blood and particle velocity for $\tau < 1$.$Pm = 0.8,\beta = 0.5,M = 0.5,Pc = 0.5,Gm = 0.5.$

**Figure 6:**
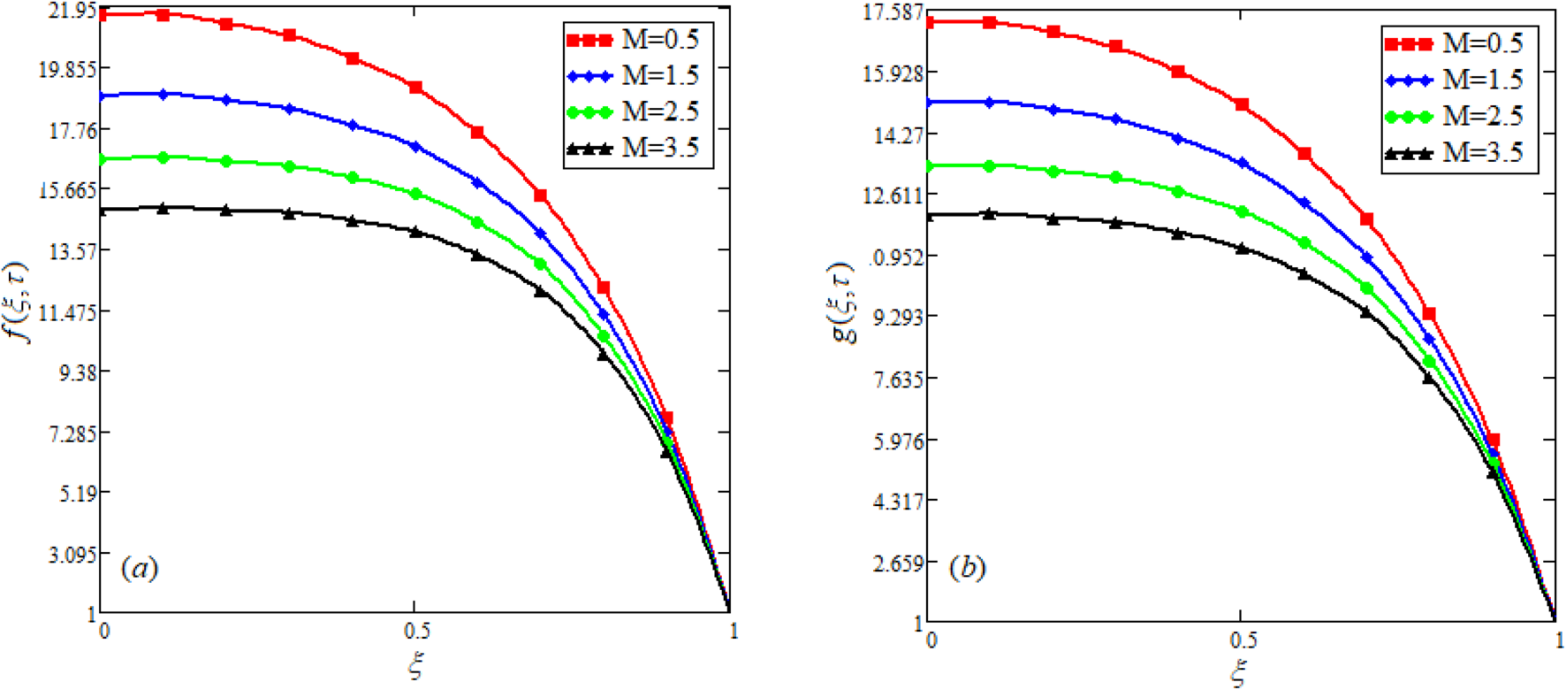
Effect of distinct values of *M* on blood and particle velocity. $M = 0.5,Pm = 0.5,\alpha = 0.5,Gm = 0.5,\beta = 0.5,Pc = 0.5.$

**Figure 7:**
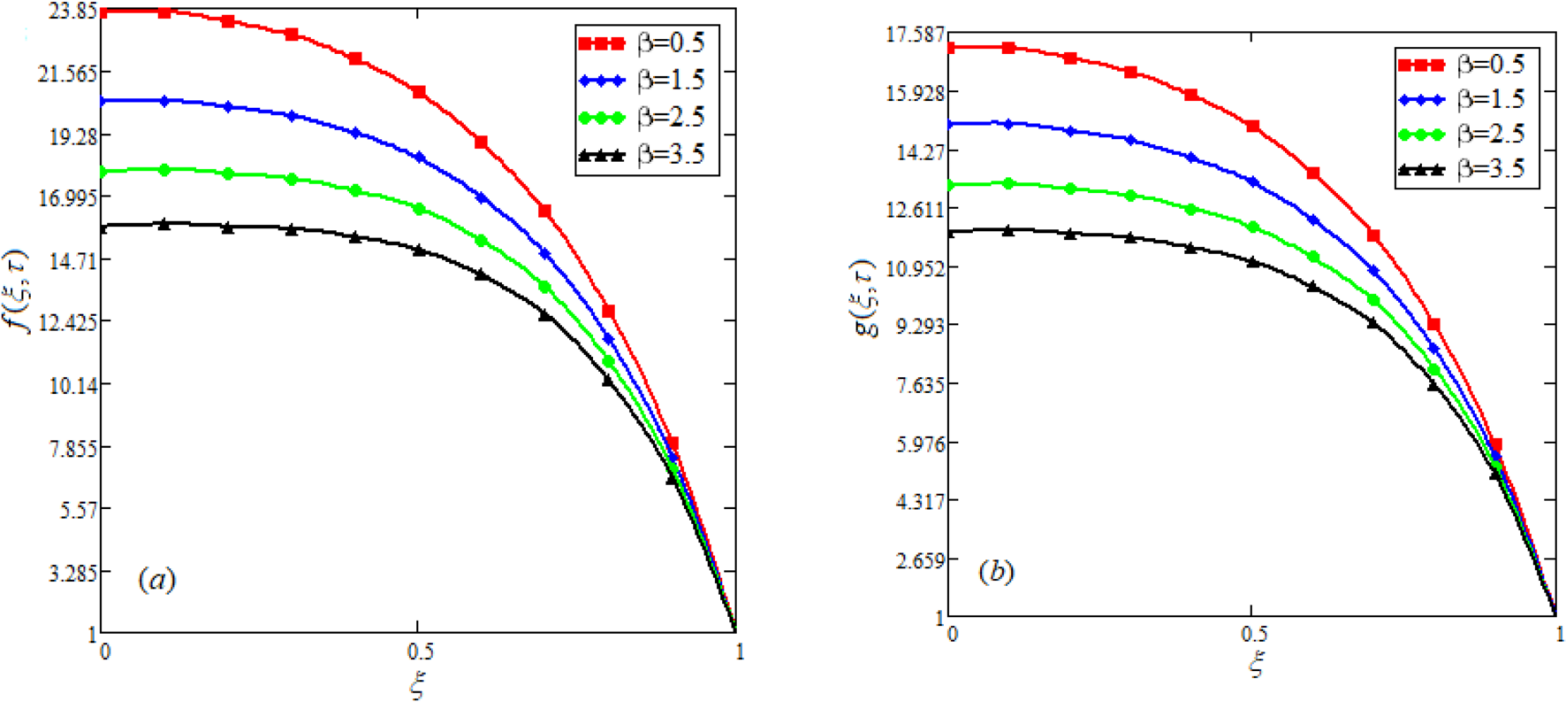
Effect of distinct values of $\beta$ on blood and particle velocity. $M = 0.5,Pm = 0.5,\alpha = 0.5,Gm = 0.5,M = 0.5,Pc = 0.5.$

**Figure 8:**
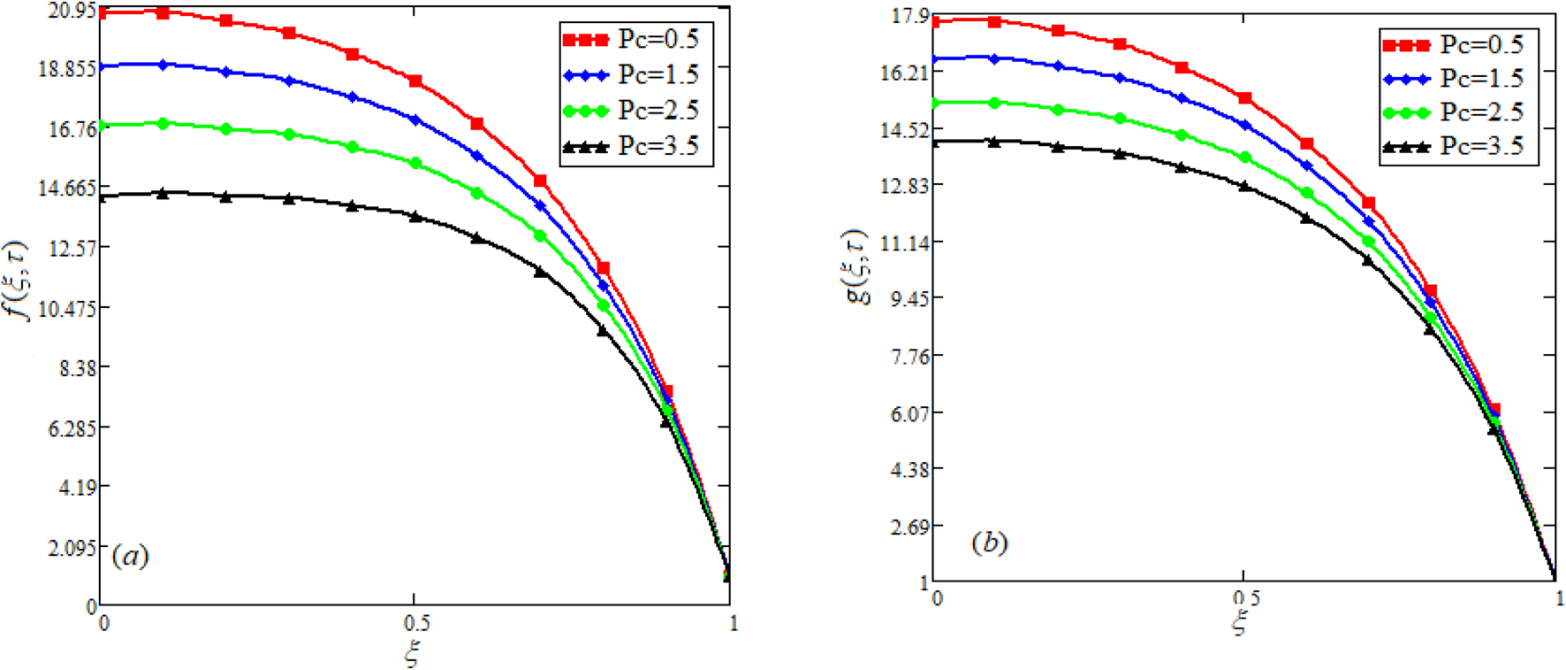
Effect of variation in *Pc* on blood and particle velocity. $M = 0.5,Pm = 0.5,\alpha = 0.5,Gm = 0.5,M = 0.5,\beta = 0.5.$

**Figure 9:**
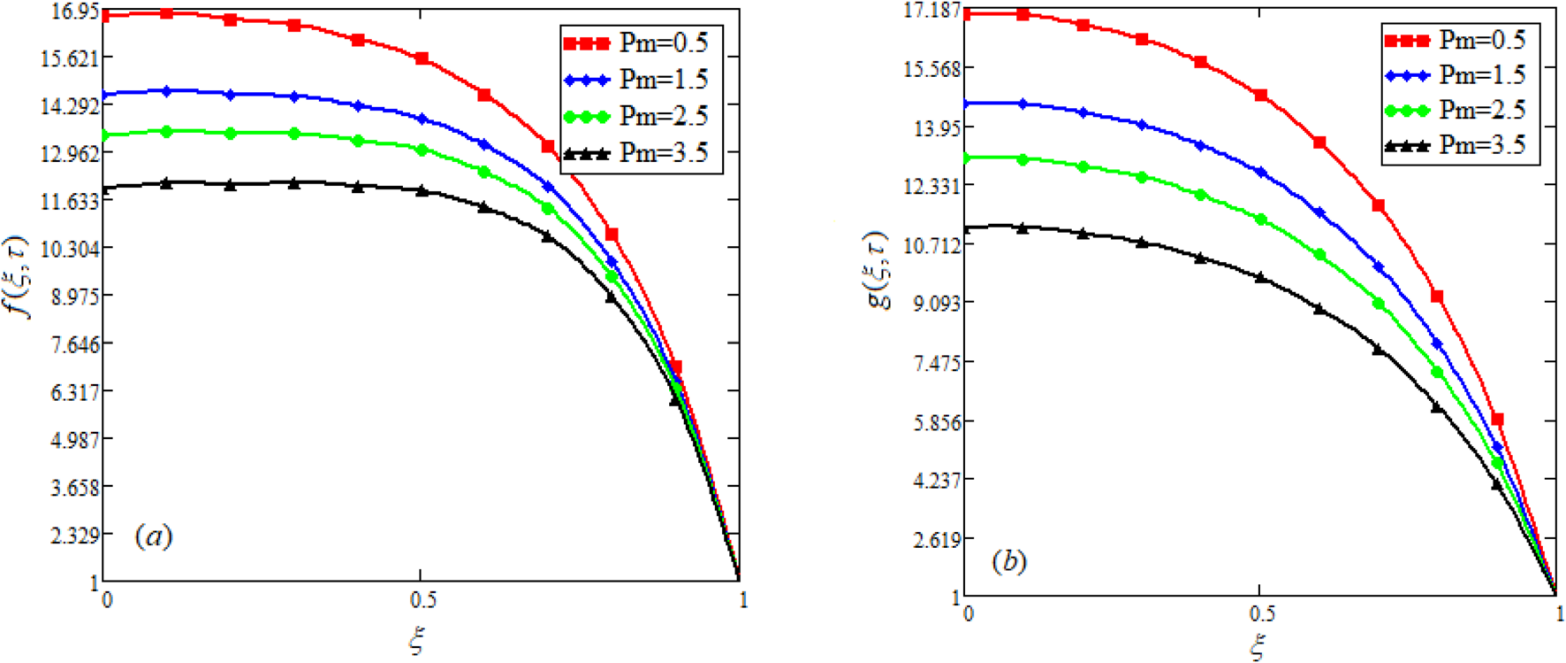
Effect of variation in $Pm$ on blood and particle velocity. $M = 0.5,Pc = 0.5,\alpha = 0.5,Gm = 0.5,M = 0.5,\beta = 0.5.$

**Figure 10:**
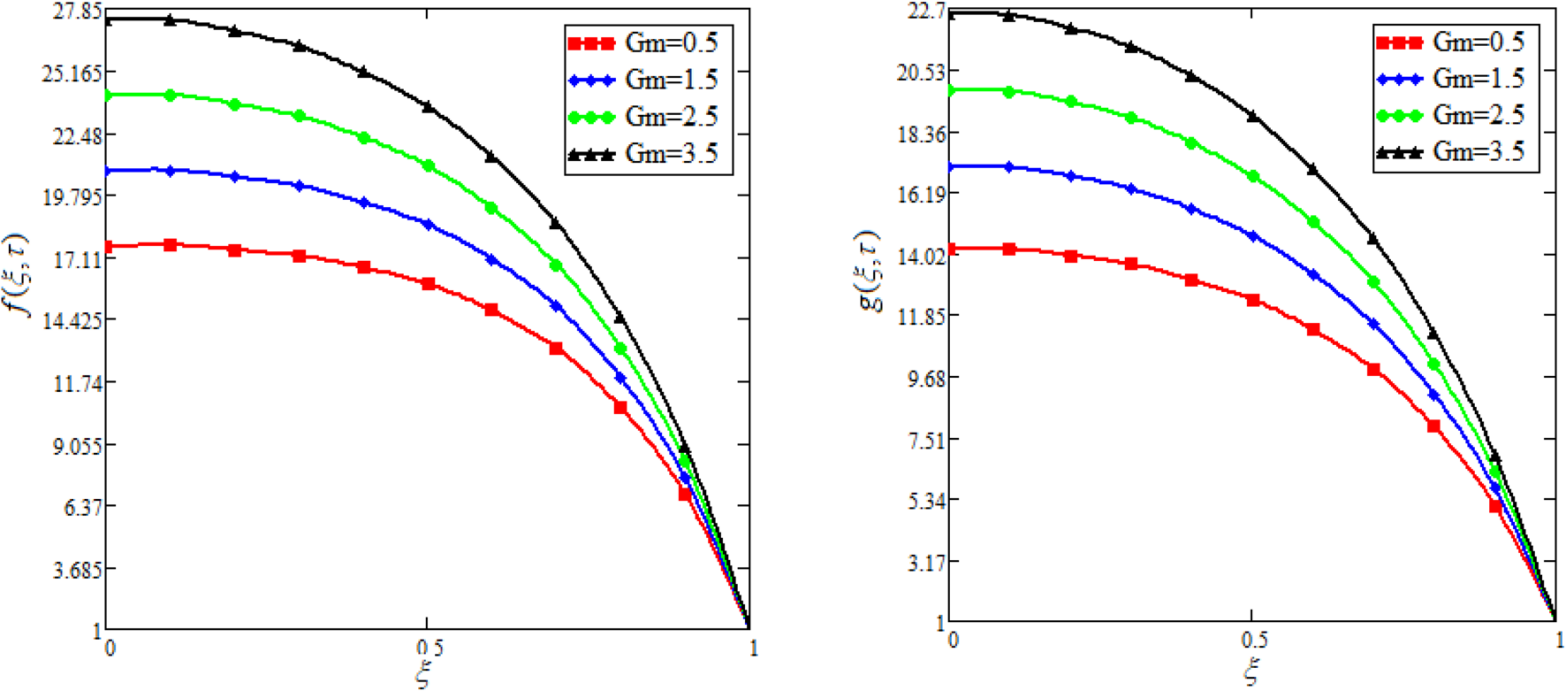
Effect of variation in $Gm$ on blood and particle velocity. $M = 0.5,Pc = 0.5,\alpha = 0.5,M = 0.5,\beta = 0.5,Pm = 0.5.$

The exact solutions for the generalized blood flow mixed with magnetic particle with joint effect of heat and mass transport are derived in this study. Various graphs are sketched to examine the flow behavior by taking fixed value for $\omega t = \frac{5\pi }{8},A_{0} = 0.5,Gr = 3.2 \times 10^{2} ,A_{1} = 0.5,$ ^39^. Figures 2, 3, 4, 5 indicates the impacts of a non-integer order parameter $\alpha$ on temperature, concentration, and velocity field. Figure 2 illustrates the variation in fluid temperature for various values of $\alpha .$ Distinct curves for temperature field are obtained at a fixed time which is termed as the memory effect. This behavior cannot be obtained using classical derivatives. The obtained curves will help the experimentalists to best fit the curve with the curve drawn from the experimental data. Moreover, when the body temperature is normal i.e. at $310K^{0} ,$ $D = 1.6 \times 10^{ - 4} mm^{2} s^{ - 1} ,$ $k = 0.52\,Jm^{ - 1} \sec^{ - 1} K^{ - 1} ,$ $\rho = 1050Kg/m^{3} ,$ $\mu = 3.2548 \times 10^{ - 3} Kg\,m^{ - 1} .\sec^{ - 1}$ $c_{p} = 3617JKg^{ - 1} K^{ - 1} ,$ For fixed value of Pr = 22.64^30^, despite of getting curves due to $\alpha ,$ a significant change in the behavior of temperature gradient is also noticed with the variation of time. The influence of $\alpha$ on concentration profile is illustrated through Fig. 3a, b by taking $Sc = 1.9 \times 10^{4}$^53^. In the graph time is also varied along $\alpha .$ It is worth noting to observe that the behavior of fractional parameter is quite opposite for larger time $(\tau > 1)$ as compared to smaller time $(\tau < 1).$ It is expected that for $\left( {\tau = 1} \right)$, the different integral curves will overlap each other. Further more, Fig. 3a, b shows various integral curves(solutions), which cannot be described by the non fractional model. These different solutions may provide space for the experimentalists to best fit their real data with one of these curves. Figures 4 and 5 are sketched for $\alpha \in (0,1)$ and $\alpha = 1,$ to investigate the effects on fluid and particle velocity. Effect of time variation is also taken into account. Different curves obtained for fractional model solution and experimentalists can find the curve which reasonably good fits to the given data. It is also noticed that for $\tau > 1$, increased values of memory parameter, both the fluid and particles velocity increased and decreased for $\tau < 1$. Figure 6 highlights the impact of magnetic parameter on both velocity profiles. It has been noticed that increased values of a magnetic parameter causes a significant decrease in fluid’s velocity. The graph clearly demonstrates that blood and particle velocity reaches its peak in the center and then steadily falls. This is because an increase in the magnetic parameter escalates the resistive forces that dominate fluid motion, decelerating the fluid and particle velocity. Anwar et al.^53^ reported a similar pattern of behavior in their investigation. These findings reveal that the intensity of the external magnetic field can be used to alter blood velocity. It is important to have a suitable external magnetic field in order to drive magnetic particles to the tumor site. Figure 7 marks the change in the blood and particle motion by variation in the Brinkman type fluid parameter $\beta$ . As can be seen in the diagram, fluid and particle motion reduces as a result of an increase in $\beta$. Physically, this is correct because the fluid's drag forces dominate and the fluid velocity falls^7^. It is obvious from the obtained result that, the adequate use of magnetic field intensity can be helpful in order to regulate the blood flow in medical field. Figures 8 and 9 are sketched to analyze the influence of particle mass parameter and particle concentration parameter on the blood and particle velocity. Same decreasing trend for velocities is noticed when the values of $Pm$ and $Pc$ are raised. The physics behind this is when particle concentration is increased the collisions of the particles also increases, due to this behavior they are dispersed from streamlines. As a result deviation from dynamic equilibrium state induces a relative velocity between the particles and the blood that generates additional energy dissipation and consequently it results in an effective viscosity^54^, consequently, fluid grows denser and thicker, slowing the flow. The variation in the values of $Pm,$ has also resulted the same behavior as observed for $Pc.$ During their research, Saqib et al.^9^ and Nandkeolyar and Das^55^ also reported this tendency .This result shows that by adjusting the values of the $Pm$ and $Pc,$ successful drug delivery to the tumor cite can be made possible. The influence of the $Gm$ on blood and particle motion is seen in Fig. 10 the obtained graph shows that both the velocities of blood and magnetic particles reduce due to an increase in mass Grashoff number.

## Conclusion

The present study briefly examined the MHD blood flow in a heated cylindrical tube. Magnetic particles are also added to the blood stream. The effects of heat and mass transfer on the flow are observed.The problem is modeled via fractional derivative. The following are some final results based on the current study:

- Caputo time fractional derivative is employed to get the solution to the problem.
- The exact solution has been obtained using Laplace and Finite Hankel transform.
- The effects of memory carrying parameter are observed.it is noticed that memory parameter gives different curves for temperature, concentration, and velocity profile of blood at constant time, but dual behavior has been observed for a long and short period of time.
- The effects of vertically applied magnetic field and different parameter has been discussed. Moreover, it has seen that magnetic field has a similar impact on blood and attractive particles velocities.
- By applying Brinkman fluid parameter, both the velocity of particles and fluid decreases.

## List of Symbols

*T*: Ambient temperature
*T*_*w*_: Surface temperature
T: Cauchy stress tensor
$f(r_{1} ,\tau )$: Dimensionless fluid velocity (ms^−1^)
$\vec{E}$: Electric field intensity
$\vec{B}$: Magnetic flux intensity
*k*_0_: Thermal conductivity of fluid (Wm^−1^ K^−1^)
*μ*_0_: Magnetic permeability
$\vec{J}$: Electric current density
*C*: Fluid’s concentration
$$C_{\infty }$$: Ambient concentration
*σ*: Electrical conductivity (s^3^ A^2^ m^−^^3^ kg^−1^)
*B*_0_: Applied magnetic field
*Pm*: Dimensionless particle mass concentration
*g*: Gravitational acceleration (ms^−^^2^)
*β*_*T*_: Thermal expansion coefficient (K^−^^1^)
*m*: Mass of magnetic particles (kg)
*N*: Number of magnetic particles
*p*: Oscillating pressure gradient
*α*: Fractional parameter
Pr: Prandtl number
*Gr*: Grashoff number
*Gm*: Mass Grashoff number
*Sc*: Schmidth number
*t*: Dimensional time
$$\mathop{V}\limits^{\rightharpoonup}$$: Velocity field.
*I*_0_: Interaction forces
$g(r1,\tau )$: Dimensionless particles velocity (ms^−1^).
*λ*_0_: Amplitude of systolic pressure gradient
$\lambda 1$: Amplitude of diastolic pressure gradient
*μ*: Dynamic viscosity of fluid (kgm^−1^ s^−^^1^)
*u(r*,*t*): Blood velocity (ms^−^^1^)
*ρ*: Density of fluid (kg m^−^^3^)
*ν*: Kinematic viscosity (m^2^ s^−^^1^)
*C*_*w*_: Wall concentration
*K*: Stokes’ constant
*β*: Brinkman-type fluid parameter
*β*_*C*_: Mass expansion coefficient
N: Number of magnetic particles
*u*_*p*_: Velocity of magnetic particles (m s^−^^1^)
*u*: Velocity of fluid (m s^−^^1^)
*Pc*: Dimensionless particle concentration parameter
*T*: Temperature field (K)
*r*: Radial axis
*M*: Magnetic parameter
*c*_*p*_: Specific heat capacity (m^2^ K^−^^1^ s^2^)
*D*: Mass diffusivity for blood
*m*: Mass of magnetic particles
